# Supplementary material for: Neuromuscular mechanisms for the fast decline in rate of force development with muscle disuse – a narrative review
Source: J Physiol. 2024 Oct 28;604(2):735–60. doi: 10.1113/JP285667 (PMC12810236; doi:10.1113/JP285667)
Supplement: Supplementary file 2 — Supplementary material [file TJP-604-735-s001.docx]

**Neuromuscular mechanisms for the fast decline of rate of force development with muscle disuse.**

Luca Ruggiero^1^ and Markus Gruber^1^

^1^Human Performance Research Centre, Department of Sports Science, University of Konstanz, Konstanz, Germany.

**SUPPLEMENTARY MATERIAL**

**S1 – Procedures used to obtain F_max_, RFD and muscle group CSA from individual studies.**

*Hvid et al. (2014).* Values of body mass were obtained from the ratio between absolute and relative (to body mass) F_max_ (from Table 1). RFD relative to body mass (Table 1) was then multiplied by body mass to obtain absolute RFD. Absolute F_max_ and RFD were then divided by mean muscle fibres CSA (digitising Figure 3) to obtain F_max_ and RFD relative to mean muscle fibres CSA.

*Monti et al. (2021) and Sarto et al. (2022).* Absolute values of F_max_, time to reach 63% F_max_ (TPF63%), and quadriceps femoris mean CSA were obtained by digitizing figures 3A, 3B, and 2G in Monti et al. (2021) and 1A, 1B, and 1F in Sarto et al. (2022). RFD was derived using F_max_ and time to reach 63% F_max_ (TPF63%) using the formula: F_max_ * 0.63 * TPF63%^-1^. RFD and F_max_ were then divided by quadriceps femoris CSA.

*Bamman et al. (1998)*. Absolute F_max_ and RFD values were retrieved (Table 3) and normalized to mean myofiber CSA values (Table 2).

*Hvid et al. (2010) and Suetta et al. (2009)*. F_max_ relative to muscle volume and time to reach 66% F_max_ (TPF66%) were retrieved from Hvid et al. (2010; Table 1 and in text, respectively). RFD was then calculated with the following formula: F_max_ relative to muscle volume * 0.66 * TPF66%^-1^.

*Kubo et al. (2000)*. Absolute values of F_max_ and RFD were retrieved (Table 2). Using the % decline of quadriceps femoris CSA (digitized from Figure 2), the % decline in F_max_ and RFD relative to quadriceps femoris CSA were derived (using formula S8; see Supplementary Material S2 of the present manuscript).

*Horstman et al. (2012)*. Values of F_max_ and RFD were retrieved for both plantarflexors and knee extensors (Table 1). No measures of CSA could be retrieved. The percentage decline of F_max_ and RFD was averaged between the two muscle groups considered.

*De Boer et al. (2007)*. Values of F_max_ and RFD were retrieved (Table 1) and normalized to quadriceps femoris CSA (Table 2).

*Valdes et al. (2020)*. Values of F_max_, RFD, and arm circumference were retrieved (Table 2). The decline of elbow flexors CSA was estimated considering arm circumference $\propto$ elbow flexor CSA^0.5^.

*Mulder et al. (2006; 2008)*. Values of F_max_ were obtained by digitizing Figure 4 from Mulder et al. (2006), while torque impulse in the first 40 ms from contraction onset relative to F_max_ was obtained by digitizing Figure 3A from Mulder et al. (2008). Torque impulse was then multiplied by F_max_, to obtain absolute values. F_max_ and torque impulse were then divided by quadriceps femoris CSA, obtained digitizing Figure 3 in Mulder et al. (2006). Of note, while F_max_ was collected before bed rest, torque impulse was collected only on day 4. Thus, values on day 4 for both F_max_ and torque impulse were used as baseline.

*Mulder et al. (2009)*. For both plantar flexors and knee extensors, F_max_ was retrieved (Table 2). The RFD relative to F_max_ was obtained by digitization of Figure 3. Absolute values of RFD were then derived multiplying by F_max_ for both muscle groups. Absolute F_max_ and RFD were then divided by plantarflexors and knee extensors CSA (Table 2). The declines in F_max_ and RFD relative to muscle CSA were then averaged between muscle groups.

*Kramer et al. (2021)*. Values of F_max_ and RFD were retrieved for both plantarflexors and knee extensors (Table 1). No measures of CSA could be retrieved. The percentage decline of F_max_ and RFD was averaged between the two muscle groups considered.

*Alkner & Tesch (2004) and Alkner et al. (2016)*. Values of F_max_ in the supine squat (knee angle: 90°) were retrieved from Alkner & Tesch (2004; Table 2). Forces at 0.1 and 0.2 s from movement initiation were obtained by digitizing Figure 2A in Alkner et al. (2016). As the method to determine contraction onset was not specified, RFD was calculated between 0.1 and 0.2 s from movement initiation, from the digitized values. Muscle volumes of quadriceps femoris and triceps surae were obtained by digitizing Figures 1 and 3 in Alkner & Tesch (2004) and were summed to obtain an estimate of lower limb muscle volume before and after bed rest. F_max_ and RFD were then divided by such estimated muscle volume to obtain relative values.

**S2 – Numerical influence of the normalisation procedure on the decline of isometric maximal and explosive strength.**

The decline of explosive strength (quantified through RFD) relative to the decline of maximal strength (quantified through F_max_) can be derived as the difference between the percentage declines (relative to baseline) of the two measures:

$$Difference in \Delta\%= \% Decline F_{max}- \% Decline RFD$$

The % decline in F_max_ and RFD can be calculated as:

$\% Decline F_{max}=\left( \frac{\frac{F_{max post}}{{CSA}_{post}}-\frac{F_{maxpre}}{{CSA}_{pre}}}{\frac{F_{maxpre}}{{CSA}_{pre}}} \right)\times100$(S1)

$\% Decline RFD=\left( \frac{\frac{{RFD}_{post}}{{CSA}_{post}}-\frac{{RFD}_{pre}}{{CSA}_{pre}}}{\frac{{RFD}_{pre}}{{CSA}_{pre}}} \right)\times100$ (S2)

Where CSA is the measure of a muscle group cross-sectional area, or a related index (see Table 1), and the subscripts *pre* and *post* indicate measures taken before and after the muscle mechanical unloading intervention, respectively. The formulae can be rearranged as:

$\% Decline F_{max}=\left( \frac{F_{maxpost}\times{CSA}_{pre}}{F_{maxpre}\times{CSA}_{post}}-1 \right)\times100$ (S3)

$\% Decline RFD=\left( \frac{{RFD}_{post}\times{CSA}_{pre}}{{RFD}_{pre}\times{CSA}_{post}}-1 \right)\times100$ (S4)

And substituting them in formula S1:

$Difference in \Delta\%=\left( \frac{F_{maxpost}\times{CSA}_{pre}}{F_{maxpre}\times{CSA}_{post}}-\frac{{RFD}_{post}\times{CSA}_{pre}}{{RFD}_{pre}\times{CSA}_{post}} \right)\times100$(S5)

And rearranging:

$Difference in \Delta\%=\left( \frac{F_{maxpost}}{F_{maxpre}}-\frac{{RFD}_{post}}{{RFD}_{pre}} \right)\times\frac{{CSA}_{pre}}{{CSA}_{post}}\times100$(S6)

The formula S6 shows that the difference in the relative change between F_max_ and RFD depends linearly on the ratio between the muscle group CSA before and after the intervention.

If only the percentage decline in muscle group CSA after the unloading intervention protocol is known (CSA Δ%), then the ratio Between CSA_pre_ and CSA_post_ can be rearranged as:

$\frac{{CSA}_{pre}}{{CSA}_{post}}= \frac{{CSA}_{pre}}{{CSA}_{pre}(1-\frac{CSA\Delta\%}{100})}=\frac{1}{1-(\frac{CSA\Delta\%}{100})}$ (S7)

And including formula S7 in formula S6:

$Difference in \Delta\%=\left( \frac{F_{maxpost}}{F_{maxpre}}-\frac{{RFD}_{post}}{{RFD}_{pre}} \right)\times\frac{{10}^{5}}{{10}^{2}-CSA\Delta\%}$ (S8)

**S3 – Figure with individual data divided by type of unloading protocol.**


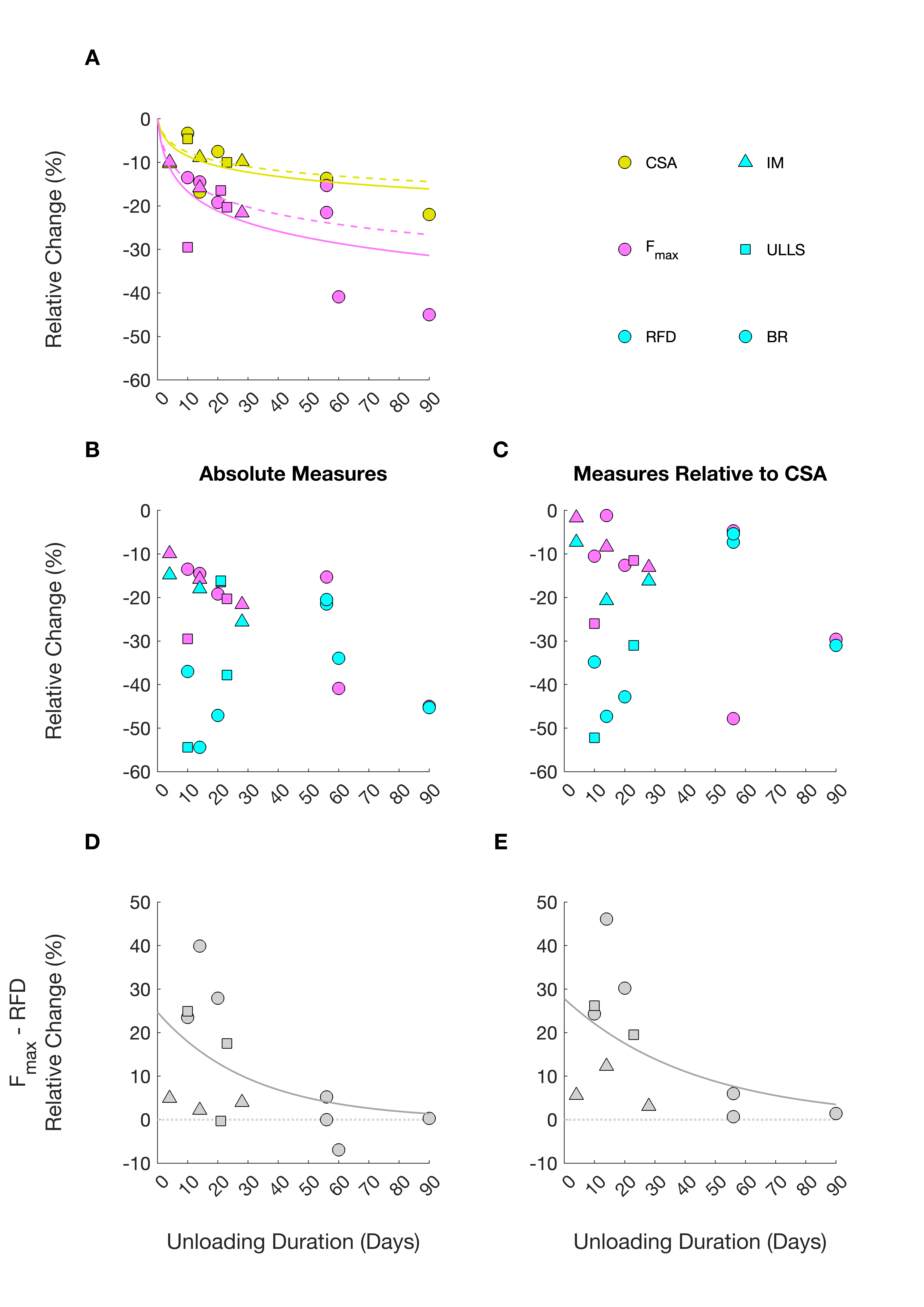


FIGURE S1 – Percent changes (relative to baseline) of muscle group cross-sectional area (CSA), isometric maximal and explosive strength (F_max_ and RFD, respectively), and difference between changes in F_max_ and RFD as function of the duration of muscle mechanical unloading. Data points are the same as in Figure 2, divided by type of unloading protocol: immobilization (IM; triangles), unilateral lower limb suspension (ULLS; squares), and bed rest (BR; circles). Refer to the description of Figure 2 in the main text for a detailed explanation.

**S4 – Hill-type force model for the simulation of the force-frequency relationship of muscle.**

To analytically verify the effect of slower twitch kinetics on peak torque and peak rate of torque development of evoked contractions, we conducted simulations (MATLAB, MathWorks, Natick, MA) with a Hill-type force model originally developed by Wexler et al. (1997), further refined by Ding et al. (2002), used to simulate the force-frequency relationship of animal and human muscles (Ding et al., 1998; Ruggiero et al., 2021). Briefly, the model reproduces muscle force as function of different number and frequency of stimuli with the following three equations:

$$C_{N} = \sum_{i=1}^{n} R_{i}\left( -\frac{t-t_{i}}{\tau_{c}} \right)\exp\left( -\frac{t-t_{i}}{\tau_{c}} \right)$$

$$R_{i}: \left( \begin{matrix} i=1 \\ i>1 \end{matrix} \right) = \left( \begin{matrix} 1 \\ 1+ \left( R_{0}-1 \right) \exp\left( -\frac{ISI}{\tau_{c}} \right) \end{matrix} \right)$$

$$\frac{dF}{dt} = A \frac{C_{N}}{K_{m}+C_{N}} - \frac{F}{\tau_{1}+ \tau_{2} \frac{C_{N}}{K_{m}+C_{N}}}$$

Where the first and second equations account for the formation of the Ca^2+^-troponin complex, and the third equation represents the development of mechanical force (or joint torque; Wexler et al., 1997). In the above formulae, *C_N_* is unitless, *n* is the number of stimuli before time *t*, *R_0_* (unitless) accounts for the nonlinear summation of the Ca^2+^ transient in muscle fibres when stimulated closely (Duchateau & Hainaut, 1986), $ISI$ is the interstimulus interval (in ms), *t_i_* is the time of the i^th^ stimulation pulse (in ms), and *τ_c_* is a time constant (in ms) controlling the rise and decay of *C_N_*, *A* (N/ms or Nm/ms) is a scaling factor for the force and the shortening velocity of the muscle, *K_m_* (unitless) is an index for the sensitivity of strongly bound cross-bridges to *C_N_*, *τ_1_* is a time constant (in ms) of force decline at the absence of strongly bound cross-bridges, and *τ_2_* is a time constant (in ms) of force decline due to the extra friction between actin and myosin resulting from the presence of strongly bound cross-bridges (Ding et al., 2002). Overall, the model is determined by the three time constants *τ_c_ , τ_1_* , *τ_2_* and by the three variables *R_0_, A, K_m_* . For the simulations herein conducted, the values of these parameters were taken from Ruggiero et al. (2021; mean values of *τ_c_ , τ_1_* , *τ_2_ , R_0_, A, K_m_* = 22 ms, 64 ms, 61 ms, 5.5, 0.3, 0.2, respectively; see “Methods” section of Ruggiero et al., 2021, for the procedures used to determine these values). Simulations were conducted to yield the same peak torque outcomes of single twitches and tetani at 10 and 100 Hz of the ankle dorsiflexors as in Ruggiero et al. (2021), represented by the black torque-time traces in Figure 4A. The values of *τ_1_* and A were then changed from 64 to 120 ms and from 0.3 to 0.22, respectively, simulating slowed twitch contraction kinetics (longer contraction and half-relaxation time by 20% and 40%, respectively) and lower peak torque (by ~18%), represented by the superimposed magenta torque-time traces in Figure 4A.

**REFERENCES**

Alkner, B.A., Norrbrand, L. & Tesch, P.A. (2016). Neuromuscular adaptations following 90 days bed rest with or without resistance exercise. *Aerosp Med Hum Perform* **87**, 610-617.

Alkner, B.A. & Tesch, P.A. (2004). Knee extensor and plantar flexor muscle size and function following 90 days of bed rest with or without resistance exercise. *Eur J Appl Physiol* **93**, 294-305.

Bamman, M.M., Clarke, M.S., Feeback, D.L., Talmadge, R.J., Stevens, B.R., Lieberman, S.A. & Greenisen, M.C. (1998). Impact of resistance exercise during bed rest on skeletal muscle sarcopenia and myosin isoform distribution. *J Appl Physiol* **84**, 157-163.

De Boer, M.D., Maganaris, C.N., Seynnes, O.R., Rennie, M.J. & Narici, M.V. (2007). Time course of muscular, neural and tendinous adaptations to 23 day unilateral lower-limb suspension in young men. *J Physiol* **583**, 1079-1091.

Ding, J., Wexler, A.S. & Binder-Macleod, S.A. (2002). A mathematical model that predicts the force-frequency relationship of human skeletal muscle. *Muscle Nerve* **26**, 477-485.

Ding, J., Binder-Macleod, S.A. & Wexler, A.S. (1998). Two-step, predictive, isometric force model tested on data from human and rat muscle. *J Appl Physiol* **85**, 2176-2189.

Duchateau, J. & Hainaut, K. (1986). Nonlinear summation of contractions in striated muscle. II. Potentiation of intracellular Ca^2+^ movements in single barnacle muscle fibres. *J Muscle Res Cell Motil* **7**, 18-24.

Horstman, A.M., De Ruiter, C.J., Van Duijnhoven, N.T.L., Hopman, M.T.E. & De Haan, A. (2012). Changes in muscle contractile characteristics and jump height following 24 days of unilateral lower limb suspension. *Eur J Appl Physiol* **112**, 135-144.

Hvid, L., Aagaard, P., Justesen, L., Bayer, L., Andersen, J.L., Ørtenblad, N., Kjaer, M. & Suetta, C. (2010). Effects of aging on muscle mechanical function and muscle fiber morphology during short-term immobilization and subsequent retraining. *J Appl Physiol* **109**, 1628-1634.

Hvid, L., Suetta, C., Nielsen, J.H., Jensen, M.M., Frandsen, U., Ørtenblad, N., Kjaer, M. & Aagaard, P. (2014). Aging impairs the recovery in mechanical muscle function following 4 days of disuse. *Exp Gerontol* **52**, 1-8.

Kramer, A., Venegas-Carro, M., Zange, J., Sies, W., Maffiuletti, N.A., Gruber, M., Degens, H., Moreno-Villanueva, M. & Mulder, E. (2021). Daily 30-min exposure to artificial gravity during 60 days of bed rest does not maintain aerobic exercise capacity but mitigates some deteriorations of muscle function: results from the AGBRESA RCT. *Eur J Appl Physiol* **121**, 2015-2026.

Kubo, K., Akima, H., Kouzaki, M., Ito, M., Kawakami, Y., Kanehisa, H. & Fukunaga, T. (2000). Changes in the elastic properties of tendon structures following 20 days bed-rest in humans. *Eur J Appl Physiol* **83**, 463-468.

Monti, E., Reggiani, C., Franchi, M.V., Toniolo, L., Sandri, M., Armani, A., Zampieri, S., Giacomello, E., Sarto, F., Sirago, G., Murgia, M., Nogara, L., Marcucci, L., Ciciliot, S., Šimunic, B., Pišot, R. & Narici, M.V. (2021). Neuromuscular junction instability and altered intracellular calcium handling as early determinants of force loss during unloading in humans. *J Physiol* **599**, 3037-3061.

Mulder, E.R., Gerrits, K.H.L., Rittweger, J., Felsenberg, D., Stegeman, D.F. & De Haan, A. (2008). Characteristics of fast voluntary and electrically evoked isometric knee extensions during 56 days of bed rest with and without exercise countermeasure. *Eur J Appl Physiol* **103**, 431-440.

Mulder, E.R., Horstman, A.M., Stegeman, D.F., De Haan, A., Belavy, D.L., Miokovic, T., Armbrecht, G., Felsenberg, D. & Gerrits, K.H. (2009). Influence of vibration resistance training on knee extensor and plantar flexor size, strength, and contractile speed characteristics after 60 days of bed rest. *J Appl Physiol* **107**, 1789-1798.

Mulder, E.R., Stegeman, D.F., Gerrits, K.H.L., Paalman, M.I., Rittweger, J., Felsenberg, D. & De Haan, A. (2006). Strength, size and activation of knee extensors followed during 8 weeks of horizontal bed rest and the influence of a countermeasure. *Eur J Appl Physiol* **97**, 706-715.

Ruggiero, L., Bruce, C.D., Streight H.B. & McNeil, C.J. (2021). Maximal results with minimal stimuli: the fewest high-frequency pulses needed to measure or model prolonged low-frequency force depression in the dorsiflexors. *J Appl Physiol* **131**, 716-728.

Sarto, F., Stashuk, D.W., Franchi, M.V., Monti, E., Zampieri, S., Valli, G., Sirago, G., Candia, J., Hartnell, L.M., Paganini, M., McPhee, J.S., De Vito, G., Ferrucci, L., Reggiani, C. & Narici, M. (2022). Effects of short-term unloading and active recovery on human motor unit properties, neuromuscular junction transmission and transcriptomic profile. *J Physiol* **600**, 4731-4751.

Suetta, C., Hvid, L.G., Justesen, L., Christensen, U., Neergaard, K., Simonsen, L., Ortenblad, N., Magnusson, S.P., Kjaer, M. & Aagaard, P. (2009). Effects of aging on human skeletal muscle after immobilization and retraining. *J Appl Physiol* **107**, 1172-1180.

Valdes, O., Ramirez, C., Perez, F., Garcia-Vicencio, S., Nosaka, K. & Penailillo, L. (2020). Contralateral effects of eccentric resistance training on immobilized arm. *Scand J Med Sci Sports* **31**, 76-90.

Wexler, A.S., Ding, J. & Binder-Macleod, S.A. (1997). A mathematical model that predicts skeletal muscle force. IEEE Trans Biomed Eng **44**, 337-348.
